# Prognostic value of C-reactive protein to albumin ratio (CAR) for mortality in older adults with sepsis: Cohort study

**DOI:** 10.1016/j.clinme.2026.100610

**Published:** 2026-07-06

**Authors:** O. Jiménez-Zarazúa, B.I. Tolentino-Pérez, L.N. Vélez-Ramírez, J.D. Mondragón

**Affiliations:** a Hospital General Zona 21 IMSS, Department of Internal Medicine, León, Guanajuato, Mexico; b Universidad de Guanajuato, Department of Medicine and Nutrition, León, Guanajuato, Mexico; c Hospital General Zona 21 IMSS, Department of Geriatrics, León, Guanajuato, Mexico; d Hospital General León, Department of Radiology, León, Guanajuato, Mexico; e University of Groningen, University Medical Center Groningen, Department of Neurology, Groningen, the Netherlands; f Universidad Nacional Autónoma de México, Instituto de Neurobiología, Departamento de Neurobiología Conductual y Cognitiva, Laboratorio de Psicofisiología, Querétaro, Mexico; g San Diego State University, Department of Psychology, Life-Span Human Senses Lab, San Diego, CA, USA

**Keywords:** C-reactive protein/albumin ratio, Older adult sepsis, Prognostic biomarker, Septic shock, 28-day mortality

## Abstract

**Study objective:**

To evaluate the prognostic value of the C-reactive protein/albumin (CAR) for mortality in older adults with sepsis.

**Design and setting:**

Prospective observational cohort study conducted at General Hospital Zone No. 21, León, Guanajuato, Mexico (Nov 2023–Aug 2025).

**Participants:**

Consecutive patients aged >60 years with Sepsis-3-defined sepsis or septic shock, excluding those with prior ICU care, transfers, malignancies, cirrhosis, autoimmune disease, or non-septic shock.

**Interventions:**

None. The CAR and Sequential Organ Failure Assessment (SOFA) scores were measured at diagnosis and 72 h.

**Main outcome measures:**

28-day all-cause mortality, with predictive performance assessed via area under the ROC curve (AUC).

**Results:**

CAR was higher in septic shock versus sepsis. In patients with sepsis, both initial (AUC = 0.867) and 72-h (AUC = 0.852) ratios predicted mortality better than SOFA (AUC = 0.786). In septic shock, SOFA was superior (AUC = 0.785 vs. 0.637). An initial ratio in the highest tertile independently predicted mortality (HR 2.88, 95% CI 1.15–5.19, p < 0.001).

**Conclusions:**

The CAR is a strong, independent mortality predictor in older adults with sepsis, outperforming SOFA in sepsis but not in septic shock. This simple biomarker can aid early identification of high-risk patients to guide timely intervention.

## Highlights

• CRP/albumin ratio predicts mortality in geriatric sepsis.
• CRP/albumin ratio outperforms SOFA risk stratification in sepsis patients.
• SOFA score remains the superior in patients with septic shock.
• Highest ratio tertile linked to 2.2-fold increased mortality risk.
• Simple biomarker reflects inflammation and physiological reserve.

## Graphical Abstract

**Figure:**
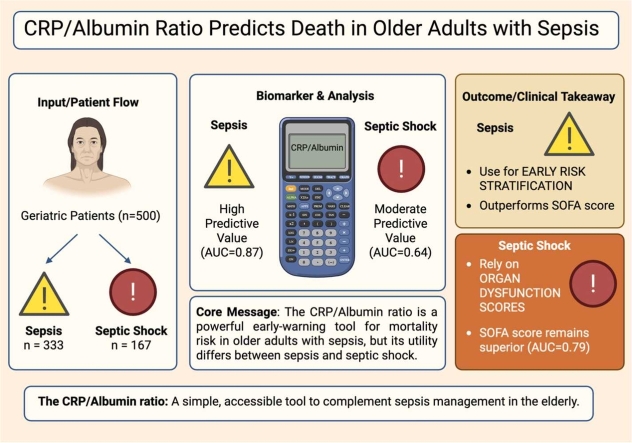

## Introduction

Sepsis remains a leading cause of global mortality and continues to place a critical burden on healthcare systems despite ongoing medical advancements.^1^ It is estimated to cause 48.9 million cases and 11 million deaths annually, accounting for nearly 20% of all global deaths.^2^ Sepsis is defined as life-threatening organ dysfunction resulting from a dysregulated host response to infection,^3^ and can progress to septic shock, which is characterised by profound circulatory and metabolic dysfunction leading to tissue hypoxia.^4^ Mortality rates remain alarmingly high, ranging from 24% to 68%,^5^, ^6^, ^7^ highlighting the urgent need for improved early detection and risk stratification strategies. This need is particularly acute in older adults, who are at increased risk due to immunosenescence (i.e., the gradual deterioration of the immune system with age) and diminished physiological reserve.^8^

Timely pathogen identification remains challenging due to the limitations of conventional culture methods, which often require more than 72 h and frequently yield false-negative results.^9^ This diagnostic delay necessitates reliable biomarkers for early intervention.^10^ While C-reactive protein (CRP) serves as a common inflammatory marker, its utility is limited by low specificity across adult populations, particularly given its elevation in various non-infectious conditions.^1^, ^11^ In contrast, albumin acts as a negative acute-phase reactant, meaning that its levels decrease in response to inflammation, and lower albumin concentrations are associated with worse outcomes and higher mortality.^12^ The CRP/albumin ratio (CAR) was first proposed by Fairclough *et al*, who demonstrated a strong correlation between CAR and the Modified Early Warning Score (MEWS) across all age groups (*r* = 0.88, *p* < 0.001).^13^ While MEWS >4 carried a relative risk of 7.8 for mortality, CAR >2 carried a relative risk of 2.6, revealing that CAR offered value in older patients, in whom MEWS performed poorly.^13^ Mechanistically, CRP reflects the intensity of the acute inflammatory response, while albumin serves as a marker of nutritional status, hepatic synthetic function and catabolic stress;^11^ their ratio therefore captures both acute inflammation and chronic physiological reserve. A meta-analysis by Liu *et al*, encompassing 3,287 patients with sepsis, confirmed the prognostic value of CAR, reporting a pooled hazard ratio of 2.1 (95% CI 1.6–2.7) for mortality.^11^ The rationale for combining CRP and albumin lies in the complementary information that they provide: CRP rises rapidly in response to infection but lacks specificity, while albumin declines more slowly and reflects underlying physiological reserve and nutritional status, together offering a more comprehensive assessment of both acute inflammatory burden and host capacity to withstand it.^14^, ^15^

In this study, we evaluate the CAR at admission and at 72 h as a predictor of clinical outcomes in older adults with sepsis and septic shock. The primary objective of this study was to evaluate the association between CAR (at admission and 72 h) and 28-day mortality in older adults with sepsis and septic shock. Secondary objectives were to: (1) compare the ratio’s discriminatory performance against SOFA, APACHE IV and lactate; (2) assess whether the ratio’s predictive value differs between sepsis and septic shock subgroups; and (3) identify independent predictors of mortality using multivariable Cox regression. We hypothesise that elevated ratios will be associated with increased 28-day mortality and worse scores on established severity indices, including the Sequential Organ Failure Assessment (SOFA) score,^16^ Acute Physiology and Chronic Health Evaluation (APACHE) IV,^17^ Charlson Comorbidity Index (CCI)^18^ and the Kirby Index,^19^ which assesses oxygenation efficiency. By validating this accessible and cost-effective biomarker, we aim to address the current gap in early risk stratification tools and ultimately improve outcomes in this high-risk population.

## Materials and methods

A prospective cohort study was conducted at General Hospital Zone No. 21 in León, Guanajuato, Mexico between November 2023 and August 2025 (22 months) to evaluate the prognostic value of the CAR in older adults hospitalised with sepsis or septic shock. Participants included patients aged >60 years; although older adult populations are commonly defined as age ≥65 years, a threshold of >60 years was selected based on the local demographic profile (higher burden of frailty and comorbidities at younger ages in this Mexican population) and consistency with prior regional sepsis studies.^1^, ^20^ While exclusion criteria, designed to ensure a homogeneous cohort and minimise confounding, comprised prior ICU care, transfer from another hospital, history of haematological or solid malignancies, liver cirrhosis, autoimmune diseases, subsequent non-septic shock or missing key data. Patients receiving IL-6 inhibitors (e.g., tocilizumab) or other biologics known to attenuate the CRP response were not identified in the screened population; however, their potential presence would constitute an additional exclusion criterion. None were excluded for this reason. These exclusion criteria were applied to minimise confounding, as each condition independently affects albumin levels and inflammatory markers: (1) prior ICU care or transfer may introduce treatment heterogeneity; (2) malignancies and cirrhosis directly alter protein synthesis and CRP production; (3) autoimmune diseases involve chronic inflammation that elevates baseline CRP. While this limits generalisability, it ensures that observed associations between CAR and mortality are attributable to acute sepsis rather than pre-existing pathology. Our stringent exclusion criteria, while methodologically necessary to minimise confounding, limit generalisability to sepsis patients with significant comorbidities. Specifically, patients with chronic liver disease, active malignancies or autoimmune conditions, populations at high risk for sepsis, were excluded. Future studies should validate CAR in these excluded groups. The study received approval from the Local Research Committee No. 1005 (R-2023-1005-052) and the National Bioethics Committee (11CEI00420190709) and was conducted in accordance with the Declaration of Helsinki and the Nuremberg Code, with strict maintenance of patient confidentiality and data protection throughout the research process.

### Demographic, paraclinical and sepsis severity scales

We systematically recorded sociodemographic, clinical and laboratory variables for detailed analysis. Baseline characteristics included gender, age, muscle mass index, vital signs and duration of hospitalisation. Prognostic evaluation used established scales such as the CCI, SOFA score, and APACHE IV. Nutritional status was assessed using the Mini Nutritional Assessment, and clinical outcomes such as discharge status and mortality were documented.

Laboratory analysis included complete blood count with leucocyte differential, lactate, and creatinine levels. Serial measurements of CRP and albumin were obtained at hospital admission and 72 h following emergency department presentation. CAR was derived by dividing the serum CRP concentration (mg/dL) by the serum albumin concentration (g/dL).^13^

### Sepsis or septic shock classification

Patients were classified as having sepsis or septic shock based on established clinical and laboratory criteria. Sepsis was defined by the presence of an infectious source and an acute increase in the SOFA score of ≥2 points attributable to infection. Septic shock was diagnosed in patients with sepsis who developed persistent haemodynamic instability, defined as a mean arterial pressure below 65 mmHg unresponsive to adequate fluid resuscitation and requiring vasopressor support, and/or a serum lactate level exceeding 2 mmol/L, in accordance with Sepsis-3 definitions.^21^

### Statistical analysis

All variables were examined for outliers and assessed for normality using the Shapiro–Wilk test. For variables approximating a normal distribution, data were summarised as mean ± standard deviation (SD); for non-normally distributed variables, data were presented as median and interquartile range (IQR). Continuous variables included CAR, APACHE IV score, SOFA score and albumin levels. Categorical variables, including mortality and gender, were summarised using frequencies and percentages with numerators and denominators reported for all percentages.

Group comparisons for the CAR were performed using one-way ANOVA for normally distributed data or the Kruskal–Wallis test for non-normally distributed data. Categorical variables were compared using chi-square tests with Fisher’s exact test used when expected cell counts were less than five. Correlations between the initial and 72-h CAR and SOFA scores were assessed using Spearman’s rank correlation due to non-normal distribution of the ratio. Correlation coefficients (ρ) were reported with 95% confidence intervals. Coefficients were interpreted as follows: very strong (0.70–1.00), strong (0.50–0.69), moderate (0.14–0.49) or weak (0.00–0.13). These categorical descriptors were used for descriptive purposes only, without predefined biological thresholds.

Survival analysis utilised the Kaplan–Meier method to estimate survival probabilities, with median survival times reported with 95% confidence intervals. Mortality distributions were compared using the Gehan–Breslow–Wilcoxon test, which gives greater weight to early events. A multivariable Cox proportional hazards regression model was employed to identify predictors of 28-day in-hospital mortality. For each predictor, hazard ratios (HRs) were reported with 95% confidence intervals and associated *p* values. The final model was developed using a backward stepwise selection procedure based on the Akaike information criterion (AIC). Assumptions of proportional hazards were verified by inspection of log-log survival plots and Schoenfeld residuals.

For the primary analysis, CAR values were categorised into tertiles (low, intermediate, high) to provide clinically interpretable risk stratification without assuming a linear relationship between CAR and mortality. Tertiles offer a straightforward means of categorising patients into clinically meaningful risk groups that are readily applicable at the bedside. To complement this approach and provide externally generalisable thresholds, we additionally identified optimal cut-off values for initial and 72-h CAR using Youden’s index, which maximises the sum of sensitivity and specificity. These cut-offs were subsequently employed in Cox proportional hazards models and sensitivity analyses. For all statistical analyses, a two-sided *p*-value <0.05 was considered statistically significant.

A significance level of α = 0.05 defined statistical significance for all primary analyses. To adjust for multiple comparisons, a Bonferroni correction was applied where indicated, with adjusted α levels reported. Missing key laboratory or outcome data were handled by complete-case analysis, as detailed in the exclusion criteria. No imputation was performed for missing data. All tests were two-tailed. The statistical software packages used were SPSS version 30 (IBM Corp., Armonk, NY, USA) and Jamovi version 2.4.11 (The Jamovi Project, Sydney, Australia).

### Study size

Based on *a priori* power calculations assuming a baseline 28-day mortality of 30%, an expected hazard ratio of 1.8 for the highest versus lowest CAR tertile, a two-sided α of 0.05%, and 80% power, a minimum of 450 patients was required. To account for potential incomplete data and attrition, we enrolled 500 patients. The target was reached after 22 months, at which point enrolment was closed.

### STROBE statement

This study was reported in accordance with the Strengthening the Reporting of Observational Studies in Epidemiology (STROBE) guidelines. A completed STROBE checklist is available as Supplementary Material.

## Results

### Study cohort and patient selection

A total of 690 patients were initially screened for inclusion in this prospective cohort study. After applying the predefined exclusion criteria, 190 patients were excluded: 92 due to histories of autoimmune disease, solid tumours or other clinically relevant comorbidities, and an additional 98 due to incomplete data or the development of non-septic shock (e.g., including 42 patients with missing serial biomarker measurements, 36 who left against medical advice, and 20 who developed cardiogenic shock) during hospitalisation. Thus**,** the final analytical cohort consisted of 500 patients, among whom 333 were diagnosed with sepsis and 167 with septic shock (Supplementary Figure 1).

### Baseline demographic and clinical characteristics

We first compared the baseline features of the included patients. Of the 500 participants, 210 (42%) were female and 290 (58%) were male. The median age was similar between the sepsis (78 years; IQR: 73–82) and septic shock (77 years; IQR: 71–82) groups. Likewise, body mass index did not significantly differ between groups, with median values of 26.4 kg/m^2^ (IQR: 22.8–31.0) in the sepsis group and 26.0 kg/m^2^ (IQR: 22.8–29.8) in the septic shock group. Nutritional status was assessed using the Mini Nutritional Assessment (MNA). In the overall cohort, the median MNA score was 19.5 (IQR 15.0–23.0), with 185 patients (37.0%) malnourished, 245 (49.0%) at risk, and 70 (14.0%) with normal nutritional status. Patients with septic shock had significantly lower MNA scores than those with sepsis (median 17.0 (IQR 12.0–21.0) vs. 21.0 (IQR 17.0–24.0), *p* < 0.001), with a higher proportion of malnutrition in the shock group (58.7% vs. 26.1%, *p* < 0.001). Detailed findings are presented in Table 1.

**Table 1:** Patient characteristics.

Variables	Overall population n = 500 median (IQR)	Sepsis n = 333 (IQR)	Septic shock n = 167 (IQR)	*p*-value
Age	78 (72–82)	78 (73–82)	77 (71–82)	0.029
Body mass index	26.2 (22.9–31)	26.4 (22.8–31–1)	26 (22.8–29.8)	0.386
Immunosuppression, n (%)	3 (0.6)	2 (0.6)	1 (0.6)	0.952
Acute Physiology and Chronic Health Evaluation IV	72 (58–86.3)	66 (55–75)	91 (77.5–109)	**<0.001**
Sequential Organ Failure Assessment	4 (2–7)	3 (2–4)	8 (6–10)	**<0.001**
Charlson Comorbidity Index	2 (1–5)	1 (1–3)	6 (4–7)	**<0.001**
Lactate mmol/L	1 (1–3)	1(1–1)	4 (3–4)	**<0.001**
Albumin initial mg/dL	3.5 (2.9–3.6)	3.5 (3.3–3.7)	2.9 (2.7–3.5)	**<0.001**
C-reactive protein initial mg/dL	40 (30–90)	39 (20–50)	90 (50–120)	**<0.001**
C-reactive protein/albumin ratio initial	12.5 (8.3–29.7)	10.5 (5.7–14.7)	31 (17.5–41.4)	**<0.001**
Albumin at 72 h mg/dL	3.1 (2.7–3.5)	3.3 (2.9–3.5)	2.8 (2.4–3.1)	**<0.001**
C-reactive protein at 72 h mg/dL	78.8 (40–140)	50 (25.5–87.9)	140 (100–188)	**<0.001**
C-reactive protein/albumin ratio at 72 h	25 (11.4–48.4)	16.7 (7.8–26.7)	50 (35.9–70)	**<0.001**
Kirby	280 (182–350)	300 (215–380)	200 (118–300)	**<0.001**
Mini Nutritional Assessment	14 (12–18.5)	14 (12–19)	13(12–18.3)	0.179
Creatinine	1.5 (0.9–3)	1.2 (0.8–2.7)	2.5 (1.5–3.8)	**<0.001**
Glucose mg/dL	127 (98.8–180)	127 (97–179)	126 (99–181)	0.675
Leucocytes 10^3^/µL	14,2 (9.7–19.6)	13 (9.3–18)	16.9 (10.8–23.3)	**<0.001**
Platelets 10^3^/µL	230 (166–313)	239 (184–312)	220 (109–325)	0.018

Among 500 patients, 333 (66.6%) had sepsis and 167 (33.4%) septic shock. The most common infection site was respiratory (191, 38.2%), followed by urinary (176, 35.2%) and soft tissue (50, 10.0%), while abdominal (17, 3.4%) and neurological (7, 1.4%) infections were less frequent. By severity, respiratory infections included 139 (27.8%) cases of sepsis and 52 (10.4%) of septic shock, and urinary infections accounted for 113 (22.6%) and 63 (12.6%), respectively; soft tissue infections comprised 38 (7.6%) sepsis and 12 (2.4%) septic shock cases, abdominal infections 10 (2.0%) and 7 (1.4%), and neurological infections 6 (1.2%) and 1 (0.2%). Mixed infections were less common, led by respiratory–urinary cases (41, 8.2%), with other combinations each representing ≤1.6% of cases. One patient (0.2%) had an unidentified infection, which was associated with septic shock (χ^2^ = 22.1; *p* = 0.009). Underlying immunosuppression, defined as chronic corticosteroid use equivalent to prednisone ≥10 mg/day for >3 months, active chemotherapy, or known HIV with CD4 < 200, was present in three patients (0.6%). All patients with active malignancies were excluded per study criteria, and no patients had HIV with CD4 < 200. The low prevalence of chronic corticosteroid use precluded inclusion of immunosuppression as a separate covariate in multivariable models.

### Illness severity and physiological markers

We next assessed markers of physiological derangement and illness severity. As anticipated, patients with septic shock presented with significantly higher APACHE IV, SOFA, and Charlson Comorbidity Index scores than those with sepsis (*p* < 0.001 for all; Supplementary Figure 2A–C). Consistent with these findings, the septic shock group also demonstrated markedly reduced Kirby scores (median 200, IQR: 118–300), as well as elevated lactate levels (median 4 mmol/L, IQR: 3–4) and higher leucocyte counts compared to the sepsis group (all *p* < 0.001; Supplementary Figure 2D, E, and L).

### Serial biomarker measurements

Given the focus of our study, we specifically analysed the dynamics of CRP, albumin, and their ratio. CRP levels were significantly higher in the septic shock group at both baseline and 72 h, whereas albumin levels were significantly lower at both time points (*p* < 0.001; Supplementary Figure 2F, G, I, and J). As a direct result of this inverse relationship, CAR was substantially elevated in septic shock patients both initially (median 31.0 vs. 10.5) and at 72 h (median 50.0 vs. 16.7; *p* ≤ 0.001; Supplementary Figure 2H and K).

### Correlation with organ failure

**Fig. 1:**
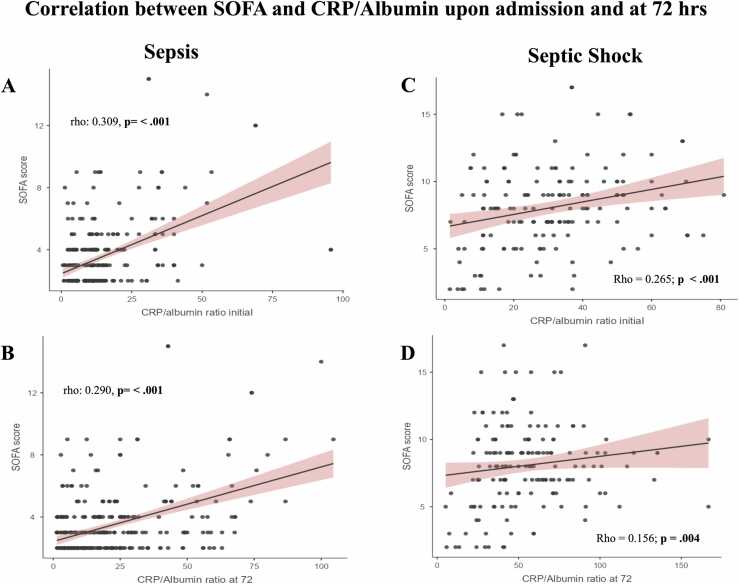
Correlation graphs in sepsis and septic shock. A) Correlation between CRP/albumin initial with SOFA score in sepsis. B) Correlation between CRP/albumin at 72 h with SOFA score in sepsis. C) Correlation between CRP/albumin initial with SOFA score in septic shock and D) Correlation between CRP/albumin at 72 h with SOFA score in septic shock.

To evaluate the clinical relevance of this ratio, we correlated it with SOFA scores. In both sepsis and septic shock groups, significant though modest correlations were observed between the SOFA score and CAR at admission (ρ = 0.309 and ρ = 0.265 respectively; *p* < 0.001) and at 72 h (ρ = 0.290, *p* < 0.001 and ρ = 0.156; *p* = 0.004; Fig. 1A–D).

### Survival outcomes and predictors

**Fig. 2:**
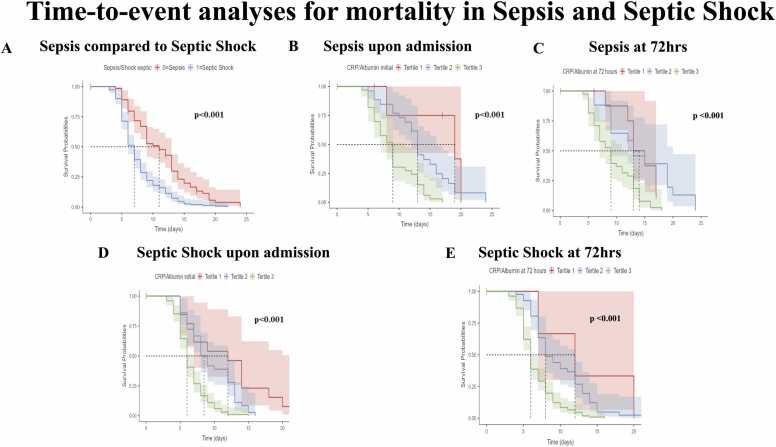
Time-to-event analyses for mortality in sepsis and septic shock. A) Sepsis versus septic shock survival analysis. B) Stratified analysis by tertiles of CRP/albumin initial in sepsis. C) Stratified analysis by tertiles of CRP/albumin at 72 h in sepsis. D) Stratified analysis by tertiles of CRP/albumin initial in septic shock and E) Stratified analysis by tertiles of CRP/albumin at 72 h in septic shock.

We subsequently examined the primary outcome of 28-day mortality. Among the 500 patients, 211 (42.2%) died. Mortality was significantly more frequent in the septic shock group (150/167, 89.8%) than in the sepsis group (61/333, 18.3%; *p* < 0.001). This disparity was reflected in the survival analysis, which showed a pronounced reduction in mean survival time for septic shock patients (7.0 ± 0.38 days) compared to sepsis patients (14.0 ± 1.7 days; log-rank χ^2^ = 28.7, *p* < 0.001; Fig. 2A).

### Stratified analysis by CAR

To further investigate the prognostic utility of the biomarker, we stratified patients by tertiles of their initial (i.e., Tertile 1, 0.5–9.81; Tertile 2, 9.82–20.6, and Tertile 3, 20.7–95.7) and 72-h (i.e., Tertile 1, 1.35–15.6; Tertile 2, 15.7–40.4, and Tertile 3, 40.5–167) CARs. In patients with sepsis, a higher initial ratio was strongly associated with reduced survival (i.e., T1, 19 days, T2, 13 days, T3, 9 days; χ^2^ = 18.7, *p* < 0.001; Fig. 2B), as well as at 72-h (i.e., T1, 14 days, T2, 13 days, T3, 9 days; χ^2^ = 16.5, *p* < 0.001; Fig. 2C). A similar pattern emerged in septic shock, where the initial ratio (T1, 12 days, T2, 8.5 days, T3, 6 days; χ^2^ = 37.7, *p* < 0.001; Fig. 2D) remained a significant predictor of mortality as well as at 72-h (T1, 12 days, T2, 8 days, T3, 6 days; χ^2^ = 27.6, *p* < 0.001; Fig. 2E).

### Multivariable regression analysis

Finally, we constructed a Cox proportional hazards model to identify independent predictors of mortality. After adjustment, the presence of septic shock (HR = 1.69, 95% CI 1.07–2.64), an initial CAR in the highest tertile (HR = 2.19, 95% CI 1.55–3.10) and elevated lactate (HR = 1.20, 95% CI 1.06–1.35) were all independently associated with increased 28-day mortality. Notably, higher creatinine (HR = 0.98, 95% CI 0.85–1.00) was not significantly associated with increased mortality. Creatinine was included as a continuous variable reflecting baseline renal function. Its lack of independent association with mortality in the fully adjusted model may be explained by the fact that acute kidney injury, while a component of multi-organ dysfunction, was largely captured by the presence of septic shock and other stronger predictors in this cohort. 72-h CAR, along with SOFA, APACHE IV, and CCI scores, did not retain independent significance (Table 2). In a *post hoc* sensitivity analysis additionally adjusting for infection source and MNA, the initial CAR in the highest tertile remained significantly associated with mortality (HR = 2.04, 95% CI 1.42–2.93, p < 0.001).

**Table 2:** COX regression model coefficients and effect sizes.

Variable			β		Wald	Sig.		HR	95.0% CI for HR	
									Inferior	Superior
Septic shock			−.524		5.286	**.021**		1.690	1.079	2.639
Acute Physiology and Chronic Health Disease Classification System IV			.001		.084	.772		1.001	.995	1.007
Sequential Organ Failure Assessment			−.034		1.900	.168		.966	.920	1.015
Charlson Comorbidity Index			−.003		.006	.937		.997	.933	1.066
CRP/albumin initial Tertile 2			.126		.187	.665		1.134	.642	2.003
CRP/albumin initial Tertile 3			−.788		19.945	**<0.001**		2.198	1.555	3.104
CRP/albumin at 72 h Tertile 2			.018		.003	.958		1.018	.518	2.002
CRP/albumin at 72 h Tertile 3			.098		.326	.568		1.103	.788	1.546
Lactate			.183		9.172	**.002**		1.200	1.067	1.351
Creatinine			.089		6.164	**.013**		1.019	.852	.981

### Discriminatory performance of biomarkers

The predictive performance was formally assessed using ROC analysis. For the entire cohort, the 72-h CAR (AUC = 0.899, 95% CI 0.868–0.930) demonstrated excellent discrimination, surpassing the initial ratio (AUC = 0.866, 95% CI 0.832–0.900) and outperforming other biomarkers like lactate (AUC = 0.812, 95% CI 0.771–0.853) and APACHE IV (AUC = 0.764, 95% CI 0.720–0.808), though it was among other strong predictors including initial CRP (AUC = 0.855, 95% CI 0.820–0.890), 72-h CRP (AUC = 0.895, 95% CI 0.864–0.926), and the SOFA score (AUC = 0.891, 95% CI 0.861–0.921). Notably, its performance varied by subgroup; it was a strong predictor in patients with sepsis (AUC = 0.852, 95% CI 0.809–0.895), a subgroup where the initial CAR (AUC = 0.867, 95% CI 0.825–0.909), initial CRP (AUC = 0.844, 95%, CI 0.799–0.889) and 72-h CRP (AUC = 0.851, 95% CI 0.807–0.895) also demonstrated excellent predictive value, but demonstrated more modest discrimination in those with septic shock (AUC = 0.682, 95% CI 0.601–0.763), where the SOFA score remained the strongest predictor (AUC = 0.785, 95% CI 0.716–0.854). This pattern of declining predictive value in the shock subgroup was consistent across all biomarkers, with lactate and APACHE IV showing particularly poor performance (AUC = 0.369, 95% CI 0.281–0.457 and 0.683, 95% CI 0.605–0.761, respectively).

In addition to tertile-based classification, optimal cut-off values for CAR were derived using Youden’s index. For the overall cohort, the optimal cut-off was 16.0 for initial CAR (sensitivity 72%, specificity 89%) and 29.4 for 72-h CAR (sensitivity 84%, specificity 87%). These thresholds demonstrated strong discrimination in ROC analysis and were independently associated with 28-day mortality in adjusted Cox models (initial CAR ≥16.0: HR 2.19, 95% CI 1.55–3.10, *p* < 0.001; 72-h CAR ≥29.4: HR 2.81, 95% CI 1.98–3.98, *p* < 0.001). The consistency of findings across both tertile-based and cut-off-based approaches supports the robustness of CAR as a prognostic marker in older adults with sepsis. A full summary of cut-off values, sensitivities, specificities and corresponding coordinates is provided in Supplementary Table 1 and illustrated in Supplementary Figure 3A–C.

## Discussion

This study evaluated the prognostic value of the CAR in older patients with sepsis and septic shock, with findings largely supporting our primary hypothesis while revealing important clinical subtleties. Our central hypothesis, that a higher CAR at admission and 72 h would be associated with increased mortality and worse clinical outcomes, was supported by the data, albeit with important nuances related to patient subgroup and timing of measurement. Notably, our results demonstrate that this simple biomarker ratio provides valuable insights into both the inflammatory response and physiological reserve in this vulnerable population.

### CAR as a composite pathophysiological indicator

A key finding emerging from our analysis is that patients with septic shock exhibited significantly higher CAR compared to those with sepsis, reflecting more severe inflammatory dysregulation. This observation aligns with the established biology of these proteins: CRP serves as a positive acute-phase reactant that increases dramatically during infection, while albumin functions as a negative acute-phase protein that declines due to hepatic reprioritisation, increased vascular permeability and oxidative stress.^11^, ^22^, ^23^ The findings support that CAR may serve as a composite biomarker reflecting both hyperinflammation (reflected by elevated CRP) and a loss of homoeostatic and nutritional reserve (reflected by declining albumin). The significantly higher ratios in the septic shock group align with the expected greater severity of the systemic inflammatory response and catabolic state in these patients. The strong, dose-dependent relationship between the initial ratio and mortality, evidenced by the tertile analysis and its independent predictive value in the Cox model (HR = 2.19 for T3), demonstrates its association with mortality. This suggests that the initial ratio, readily available at admission, is associated with higher mortality and may reflect intense inflammation and poor physiological reserve. Importantly, in older adults, this ratio may reflect both acute inflammatory status and underlying chronic vulnerability factors including frailty, malnutrition, and comorbidities, all known to significantly influence sepsis outcomes.^1^

### Timing and contextual considerations in prognostic utility

Analysis of temporal patterns revealed that the initial CAR had superior predictive value compared to the 72-h measurement, particularly in sepsis patients (AUC 0.867 vs. 0.852). This suggests that the ratio is most useful for early risk stratification in sepsis patients. In contrast, the reduced predictive power of the ratio in established septic shock indicates that, once significant organ dysfunction is present, the extent of organ failure, as measured by the SOFA score, becomes a more important predictor of outcome than the initial inflammatory response. This interpretation is supported by previous reports showing that while CRP dynamics may correlate with treatment response, their association with sepsis severity and mortality is variable.^24^, ^25^

### Comparative performance against established markers and prior studies

Our findings extend three key prior studies of the CAR in older adult populations. Ayranci *et al* reported that CAR predicted in-hospital mortality in emergency department patients (AUC 0.723) and demonstrated that combining CAR with NLR improved predictive power.^26^ Cha *et al* found that CAR alone predicted 28-day mortality in older sepsis patients (AUC 0.747) and, when combined with premorbid ambulation ability, was not inferior to SOFA.^27^ Hazar *et al* enrolled 1,971 much older patients with acute respiratory diseases and reported a CAR AUC of 0.84 for hospital mortality.^28^ Our prospective study demonstrates substantially higher CAR values (sepsis median 10.5, septic shock median 31.0) and superior discriminatory performance (initial CAR AUC 0.866, 72-h CAR AUC 0.899) compared to these prior retrospective reports.

Several factors likely explain these differences. First, our prospective design ensured standardised timing of laboratory measurements, complete data capture and uniformly applied exclusion criteria, whereas retrospective studies are subject to selection bias and variability in measurement timing. Second, our cohort included a higher proportion of septic shock patients (33.4%) with a correspondingly higher 28-day mortality rate (42.2%), explaining why our optimal CAR cut-offs (initial 16.0, 72-h 29.4) exceed those previously reported. Third, our serial measurements at 72 h, a novel contribution not present in prior studies, allowed us to capture dynamic inflammatory responses, with the 72-h CAR (AUC 0.899) outperforming both the initial CAR and SOFA in the overall cohort. Fourth, our subgroup analysis by shock status revealed that CAR performance varies substantially across illness severity: it demonstrated excellent discrimination in sepsis (AUC 0.867, outperforming SOFA) but more modest performance in established septic shock (AUC 0.682), where SOFA remained superior (AUC 0.785), a nuanced finding not previously reported. In our overall cohort, the 72-h CAR (AUC 0.899) outperformed lactate (0.812), APACHE IV (0.764), and even SOFA (0.891); however, this superiority was not uniform across subgroups. In the septic shock subgroup, SOFA became the most accurate predictor (0.785) while CAR declined to 0.682, suggesting that once multi-organ failure is established, SOFA captures the dominant prognostic factor. The sustained superiority of SOFA in predicting outcomes in septic shock patients reaffirms that organ dysfunction scores remain essential for prognosis in the most critically ill, where multi-organ failure becomes the primary mortality driver.^29^ Collectively, our findings indicate that CAR is most useful for early risk stratification before advanced organ failure develops, whereas traditional organ dysfunction scores remain preferred once septic shock is established.

From a health economic perspective, CAR offers a cost-effective alternative to more resource-intensive prognostic tools. Unlike APACHE IV or SOFA, which require the aggregation of multiple clinical and laboratory variables and dedicated personnel time, CAR is derived from two widely available, inexpensive assays that are routinely obtained in the evaluation of septic patients. This simplicity, particularly in resource-limited settings where access to advanced scoring systems or costly biomarkers such as procalcitonin may be constrained, enhances the clinical utility of CAR as a practical tool for early risk stratification. Furthermore, the trajectory of CAR over time provides important prognostic information beyond the initial value. In our cohort, the 72-h CAR (AUC 0.899) outperformed the admission CAR (AUC 0.866), indicating that the dynamic inflammatory response to treatment adds discriminatory value. Patients whose CAR increased or failed to decrease by at least 30% at 72 h had significantly higher 28-day mortality (HR 2.34, 95% CI 1.67–3.28, *p* < 0.001) compared to those with a sustained reduction. This finding suggests that serial CAR measurements can aid in identifying patients who are not responding adequately to initial management and may benefit from intensified therapy or closer monitoring.

The use of optimal cut-offs derived from Youden’s index (admission CAR ≥16.0, 72-h CAR ≥29.4) provides clinically actionable thresholds that complement the tertile-based risk stratification. These cut-offs demonstrated strong discrimination in the overall cohort and were independently associated with mortality in adjusted Cox models. The consistency of findings across both tertile-based and cut-off-based approaches supports the robustness of CAR as a prognostic marker in older adults with sepsis.

### Limitations and future research directions

Our study addresses key limitations of prior work: unlike Ayranci *et al*, we focused on a homogeneous sepsis population; unlike Cha *et al*, we included 33.4% septic shock patients; and unlike Hazar *et al*, we included all infection sources, supporting CAR’s generalisability. However, each prior study has unique strengths that we could not match: Cha *et al*’s multicentre design, Hazar *et al*’s large sample size (n = 1,971), and Ayranci *et al*’s exploration of combined markers (CAR + NLR).

This study has several limitations. First, as a single-centre investigation, our findings may lack generalisability. Second, exclusion of participants with incomplete data may have introduced selection bias. Third, we could not correlate CAR with inflammatory markers such as IL-1, IL-6, TNF-, or procalcitonin, which would have strengthened pathophysiological interpretation. Fourth, the high prevalence of malnutrition in our cohort may have influenced albumin levels and CAR interpretation. Fifth, our tertile-based classification requires validation through established clinical cut-points.

The high prevalence of malnutrition in our cohort (as reflected by low MNA scores) may have influenced albumin levels and consequently affected CAR interpretation. However, our sensitivity analysis adjusting for nutritional status confirmed that CAR remained independently associated with mortality, suggesting that the ratio captures both the inflammatory response to infection and the underlying nutritional reserve, a combined measure that may be particularly informative in older adult populations where malnutrition is common.

Despite these limitations, CAR is an accessible, cost-effective biomarker that is associated with mortality in older adults with sepsis. The advanced age of our participants (median >77 years) heightens clinical relevance, as immunosenescence and frailty magnify the ratio’s biological significance. An elevated CAR likely reflects not only acute infection but also reveals underlying physiological vulnerability, explaining its superior predictive performance over the Charlson Comorbidity Index.

Based on these observations, the initial CAR is associated with higher risk in older adults with sepsis in the emergency department. Future multicentre studies should validate these results across diverse populations. Subsequent research should explore whether ratio-guided treatment strategies, including antibiotic timing, nutritional support, resuscitation or anti-inflammatory approaches, can improve survival. Finally, investigations correlating CAR with cytokine and procalcitonin measurement would further elucidate its mechanisms and optimise clinical application.

## Conclusion

In conclusion, this study provides evidence that CAR is a readily available prognostic biomarker in older adults with sepsis. The initial ratio is a strong independent predictor of 28-day mortality, while the 72-h ratio offers superior discrimination in sepsis patients specifically. These findings underscore the interplay between acute inflammation and chronic physiological reserve in determining outcomes. CAR may capture this interplay and is associated with mortality in older adults with sepsis and septic shock.

## CRediT authorship contribution statement

**J.D. Mondragón:** Writing – review & editing, Writing – original draft, Visualization, Validation, Supervision, Software, Project administration, Methodology, Investigation, Formal analysis, Conceptualization. **L.N. Vélez-Ramírez:** Writing – review & editing, Validation, Software, Investigation, Data curation. **B.I. Tolentino-Pérez:** Writing – review & editing, Methodology, Investigation, Conceptualization. **O. Jiménez-Zarazúa:** Writing – review & editing, Writing – original draft, Validation, Supervision, Project administration, Methodology, Investigation, Funding acquisition, Formal analysis, Data curation, Conceptualization.

## Ethics approval and consent to participate

Prior to conducting the research, the protocol was approved by the Local Research Committee No. 1005 (approval number, R-2023-1005-052) and the National Bioethics Committee of Instituto Mexicano del Seguro Social (approval number, 11CEI00420190709). Patients and/or their family members or legal representatives signed an informed consent form to participate in the study. The clinical and laboratory data of interest were anonymised.

## Funding

This study was supported by a grant from 10.13039/501100003141CONACYT (Grant #2022-000018-02NACF-12766) and received no commercial funding.

## Declaration of competing interest

The authors declare that they have no known competing financial interests or personal relationships that could have appeared to influence the work reported in this paper.

## Data Availability

The data that support the findings of this study are not publicly available due to privacy and ethical restrictions, as they contain sensitive clinical information from a vulnerable geriatric population. De-identified data may be made available upon reasonable request to the corresponding author, subject to approval by the relevant institutional ethics committees at General Hospital Zone No. 21 IMSS and the National Bioethics Committee of Mexico.

## Footnotes

Appendix A Supplementary data associated with this article can be found in the online version at doi:10.1016/j.clinme.2026.100610.
