# Supplementary material for: Prognostic value of C-reactive protein to albumin ratio (CAR) for mortality in older adults with sepsis: Cohort study
Source: Clin Med (Lond). 2026 Jul 6;26(5):100610. doi: 10.1016/j.clinme.2026.100610 (PMC13396719; doi:10.1016/j.clinme.2026.100610)
Supplement: Supplementary file 1 — Supplementary material [file mmc1.docx]

**Supplementary Material**

**Supplementary Table 1.** Area under the curve for receiver operating characteristic analyses

|  | Mortality (n =211 ) | | | |
| --- | --- | --- | --- | --- |
| Population | **Biomarker** | **AUC (95% CI)** | **Cut-offs** | **Coordinates** |
| Overall (n=211) | Acute Physiology and Chronic Health Evaluation IV | **.764 (.720–.808)** | 77 (score) | (.65, .79) |
|  | Sequential Organ Failure Assessment | **.891 (.861–.921)** | 5.0 (score) | (.81, .86) |
|  | Lactate | **.812 (.771–.853)** | 1.5 (mmol/L) | (.71, .90) |
|  | C-reactive protein initial | **.855 (.820–.890)** | 50 (mg/dL) | (.79, .87) |
|  | C-reactive protein at 72 hours | **.895 (.864–.926)** | 90 (mg/dL) | (.85, .85) |
|  | C-reactive protein / albumin ratio initial | **.866 (.832–.900)** | 16.0 | (.72, .89) |
|  | C-reactive protein/ albumin ratio at 72 hours | **.899 (.868–.930)** | 29.4 | (.84, .87) |
| Sepsis (n=61) | Acute Physiology and Chronic Health Evaluation IV | .598 (.522–.674) | 77 | (.34, .81) |
|  | Sequential Organ Failure Assessment | **.786 (.724–.848)** | 4.0 | (.72, .74) |
|  | Lactate | .549 (.470–.628) | 1.5 | (.14, .95) |
|  | C-reactive protein initial | **.844 (.799–.889)** | 50 | (.77, .83) |
|  | C-reactive protein at 72 hours | **.851 (.807–.895)** | 89.4 | (.75, .87) |
|  | C-reactive protein / albumin ratio initial | **.867 (.825-.909)** | 16 | (.73, .90) |
|  | C-reactive protein/ albumin ratio at 72 hours | **.852 (.807-.897)** | 29.4 | (.73, .89) |
| Shock (n=150) | Acute Physiology and Chronic Health Evaluation IV | **.683 (.605-.761)** | 82 | (.70, .64) |
|  | Sequential Organ Failure Assessment | **.785 (.716-.854)** | 6.0 | (.85, .64) |
|  | Lactate | .369 (.281-.457) | 2 | (.94, .50) |
|  | C-reactive protein initial | .647 (.564-.730) | 45 | (.84, .41) |
|  | C-reactive protein at 72 hours | .675 (.595-.755) | 95 | (.84, .52) |
|  | C-reactive protein/ albumin ratio at 72 hours | .682 (.553-.721) | 29.9 | (.88, .58) |
|  | C-reactive protein / albumin ratio initial | .637 (.601-.763) | 17.3 | (.78, .52) |

**Supplementary Figure 1.** Patient selection flow diagram


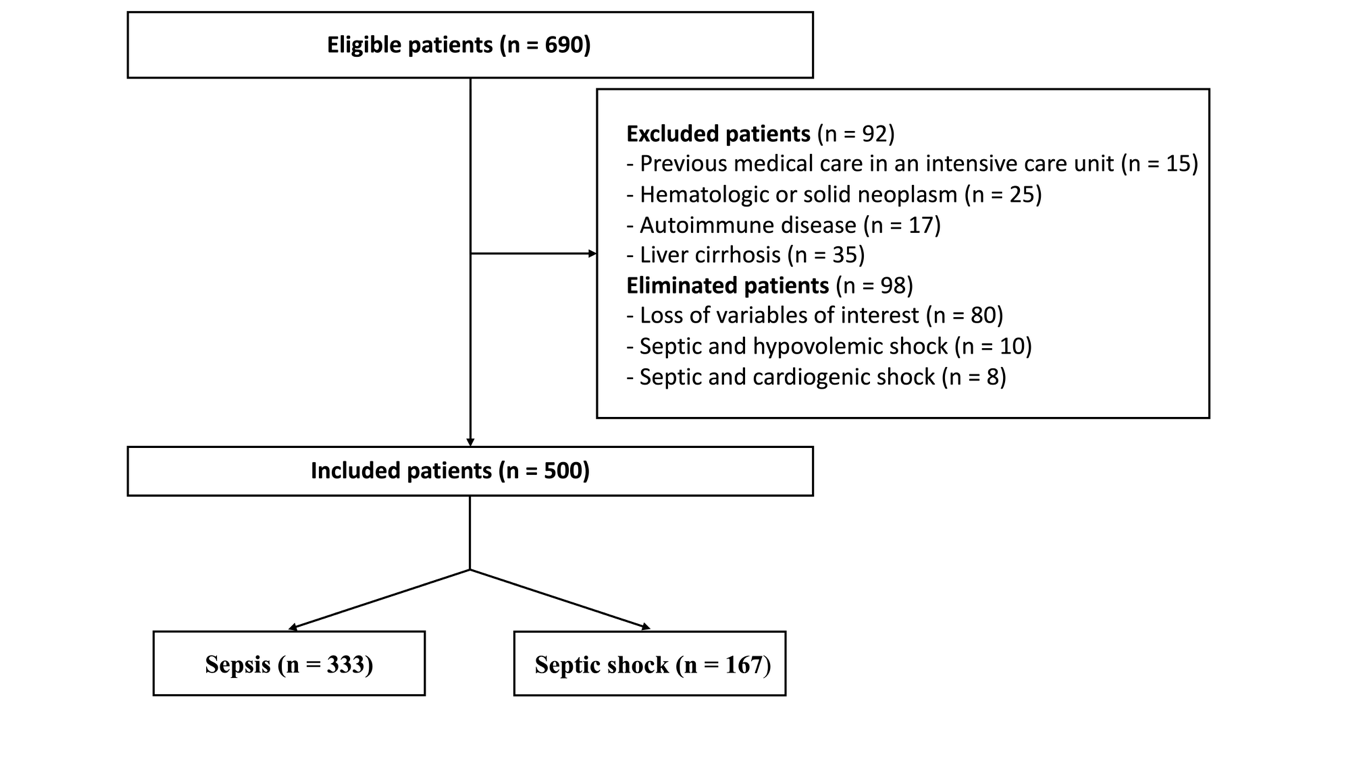
Patient selection flow diagram. Patient selection flow diagram displaying a description of the patients included for data analysis, as well as a description of the patients excluded and eliminated from statistical analyses.

**Supplementary Figure 2.** Sepsis and Septic Shock according to biomarkers.

Sepsis and Septic Shock according to biomarkers. Box-plots of the different biomarkers evaluated. **A)** APACHE IV score; **B)** SOFA score; **C)** Charlson Comorbidity index; **D)** Kirby Index; **E)** Lactate serum levels; **F)** CRP serum initial levels; **G)** Albumin initial serum levels; **H)** CRP/albumin initial levels; **I)** CRP at 72 hours levels; **J) )** Albumin at 72 hours serum levels; **K)** CRP/albumin at 72 hours levels; and **L)** Leukocytes levels.

**Supplementary Figure 3.** Receiver operating characteristic curves of different biomarkers for predicting mortality in Sepsis and Septic Shock.

Receiver operating characteristic curves of different biomarkers for predicting mortality. **A)** in Sepsis and **B)** in Septic Shock.
